# A Raft-Associated Species of Phosphatidylethanolamine Interacts with Cholesterol Comparably to Sphingomyelin. A Langmuir-Blodgett Monolayer Study

**DOI:** 10.1371/journal.pone.0005053

**Published:** 2009-03-30

**Authors:** Michal Grzybek, Jakub Kubiak, Agnieszka Łach, Magdalena Przybyło, Aleksander F. Sikorski

**Affiliations:** 1 Laboratory of Cytobiochemistry, Biotechnology Faculty, University of Wrocław, Wroclaw, Poland; 2 Academic Centre for Biotechnology of Lipid Aggregates, Wroclaw, Poland; University of Geneva, Switzerland

## Abstract

**Background:**

Specific interactions between sphingomyelin (SM) and cholesterol (Ch) are commonly believed to play a key role in the formation of rafts in the biological membranes. A weakness of this model is the implication that these microdomains are confined to the outer bilayer leaflet. The cytoplasmic leaflet, which contains the bulk of phosphatidylethanolamine (PE), phosphatidylserine (PS) and phosphatidylinositol (PI), is thought also to harbour half of the membrane cholesterol. Moreover, SLPE (1-stearoyl-2-linoleoyl-sn-glycero-3-phosphatidyl-ethanolamine) has recently been shown to be enriched in isolated detergent-resistant membranes (DRM), and this enrichment was independent of the method of isolation of DRM.

**Methodology/Principal Findings:**

Here we present quantitative evidence coming from Langmuir-Blodgett monolayer experiments that SLPE forms complex with Ch similar to that between SM and Ch. The energies of these interactions as calculated form the monolayer studies are highly negative. FRAP analysis showed that NBD-Ch recovery was similar in liposomes composed of DOPC/Ch SM or SLPE but not DPPE, providing further evidence that SLPE may form an l_o_ phase in the presence of high Ch concentration. Experiments on the solubility of DOPC liposomes containing DPPE/Ch (1∶1), SM/Ch (1∶1) or SLPE/Ch (1∶1) showed the presence of Triton X-100 insoluble floating fraction (TIFF) in the case of SM/Ch or SLPE/Ch but not in DPPE/Ch containing liposomes. Quantitative determination of particular lipid species in the TIFF fraction confirms the conclusion that SLPE (or similar PE species) could be an important constituent of the inner leaflet raft.

**Conclusion:**

Such interactions suggest a possible existence of inner-leaflet nanoscale assemblies composed of cholesterol complexes with SLPE or similar unsaturated PE species.

## Introduction

The mammalian plasma membrane, is made up of many types of lipids. These lipids fall into three main groups: glycerophospholipids, sphingolipids and cholesterol. It is known that they are distributed heterogeneously between the outer and inner membrane leaflets. The former comprises mainly phosphatidylcholine and sphingolipids, while the latter contains the bulk of the membrane's phosphatidylserine (PS), phosphatidylinositol (PI) and phosphatidylethanolamine (PE). Cholesterol is thought to be distributed equally between the leaflets. It has been suggested before but recently become apparent that the lipids are also nonuniformly distributed laterally [1]. Membrane microdomains, called lipid rafts, are distinct in their lipid and protein contents from the bulk of the membrane [2], [3], [4]. The enrichment of cholesterol and sphingomyelin in lipid rafts has prompted many studies on the interactions between these two principal raft components [5], [6], [7], [8], [9], [10], [11], [12], [13], [14]. Membrane rafts are suggested to be transient, driven by interactions between specific lipids that can be clustered under certain conditions [15], [16]. Most of the evidence from lipid-lipid interactions comes from model systems and the reconstituted membrane rafts models suggest that these are resistant to cold Triton X-100 extraction, in consequence probably of their presumed liquid ordered state (l_o_) state. The accepted model of l_o_ phase assumes that sphingolipids are tightly packed, but nevertheless display high lateral mobility. This type of phase is preferred when the acyl chains are mostly saturated and there is a high content of cholesterol. Sphingolipids contain mainly long saturated or monounsaturated acyl chains that allow them to pack tightly together. Domains of SM/Ch exist in model systems, where they undergo phase separation from the fluid disordered phase [7], [12], [13], [14]. A raft model, based on the SM and Ch interactions may explain the formation of microdomains in the outer bilayer leaflet, since it consists predominantly of sphingomyelin, cholesterol and phosphatidylcholine with mainly polyunsaturated acyl chains [13], [17]. However, it offers little explanation for the presumed presence of lipid domains in the cytoplasmic bilayer leaflet. Because of the resistance to Triton X-100 extraction lipid rafts are isolated by ultracentrifugation in a density gradient, and the isolated fractions are called detergent-resistant membranes (DRM). Although there is much evidence to suggest that DRM are related to lipid rafts it should be noted that these two terms are not synonymous and that not all data obtained on DRM need pertain equally to lipid rafts [18], [19]. Recent data on lipidomics of DRM reveal that in addition to abundant sphingomyelin and cholesterol they contain other phospholipids, mostly with fully saturated or monounsaturated acyl chains. Predominant among these are the phosphatidylethanolamine glycerophospholipids and plasmalogens [20], [21], [22], [23], [24]. Phosphatidylserine, which is a relatively minor membrane component, is three times more prevalent in DRM than in the bulk plasma membrane, while phosphatidylinositols are rather diminished, as are phosphatidylcholine species [20]. As PE, PS are mainly confined to the cytoplasmic half of the membrane, it is thus of interest to determine how the inner leaflet lipids are organised and what rules govern their partitioning into lipid rafts. Theoretical models suggest that: (i) outer-leaflet rafts induce the formation of inner-leaflet domains which could then sequester acylated proteins; (ii) the association of internal peripheral membrane proteins with transmembrane raft-linked proteins may cause their detergent resistance; and finally, (iii) there are only outer-leaflet rafts and the insolubility of certain proteins is fortituous and independent of lipid association [22]. Because PEs are the most abundant phospholipids found in the inner bilayer leaflet, we have searched for their ability to form specific complexes with cholesterol. Although Ch is known to induce domain formation in PE/Ch mixed monolayers, the interactions between PE and Ch seem to depend on the level of saturation of PE acyl chains [25], [26], [27], [28], [29]. In DPPE/Ch mixtures, the strong PE-PE interactions minimize mixing with Ch and result in the formation of Ch-rich lipid domains [25]. PEs occur in the membrane predominantly as sn-1 saturated sn-2 unsaturated lipids, and recent data show that some DRM preparations are enriched in 1-stearoyl-2-linoleoyl-sn-glycero-3-phosphoethanolamine (SLPE), regardless of the method of isolation [20]. It was interesting to look at the possibility of interaction of this lipid with cholesterol as possible molecular basis of raft formation in the inner membrane monolayer. In this paper we present data on the interactions between SLPE and Ch and the formation of condensed phases of these two lipids. By studying the interactions by the Langmuir-Blodgett technique at the air-water interface, we have found that SLPE/Ch interactions are strongly attractive and comparable to those recorded for the SM/Ch. We suggest therefore that interaction of mono- and di-unsaturated PE species with cholesterol could be molecular basis of condensed phase formation.

## Results

### The interactions between Cholesterol and SLPE are comparable to those between Cholesterol and SM

**Figure 1:**
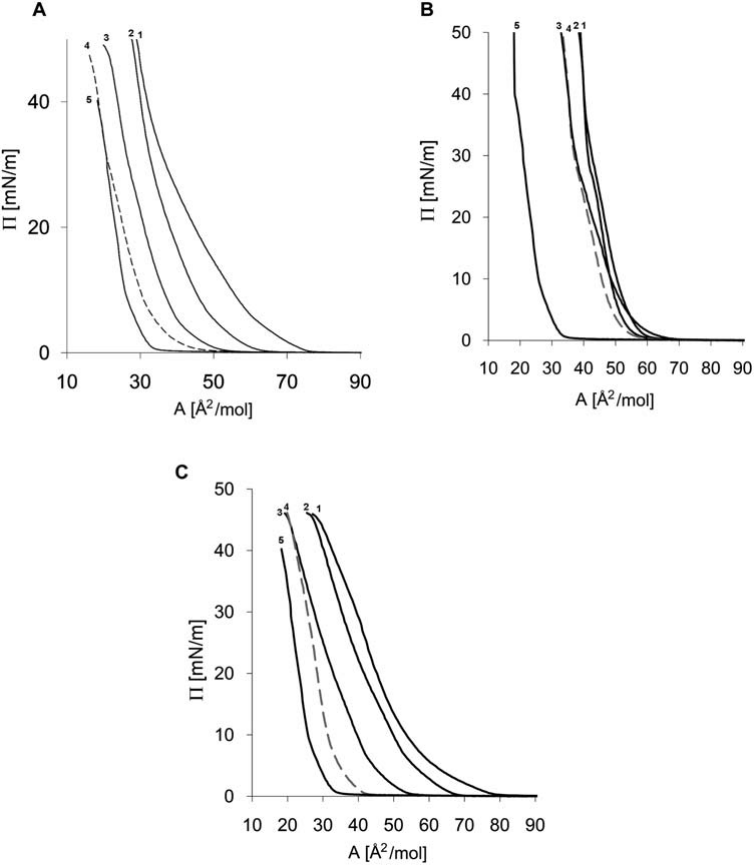
Examples of recorded Π-A isotherms obtained at 25°C. Pure phospholipids(1), cholesterol (5) and 4∶1(2), 2∶1(3) and 1∶1(4) lipid/Ch mixtures. (A) SM/Ch, (B) DPPE/Ch and (C) SLPE/Ch.

The isotherms of surface pressure versus area of pure lipids and their mixtures with cholesterol are shown in Figure 1. To quantify the effect of Ch on the other lipids, the average molecular area of an ideal mixture at 3 mN/m was calculated and compared to the observed molecular area. Low surface pressure values are commonly used in studies of miscibility, whereas high surface pressure conditions are preferred for simulation of biological membrane densities [13], [30]. The average of the different monolayers was taken from the 5–7 most consistent isotherms from 3 independent monolayers for each lipid and lipid/Ch mixture. As shown in Table 1, Ch had a compressing effect on SM but caused an expansion of the DPPE. The degree of contraction of SM increased with increasing Ch content and reached 23% for 1∶1 SM/Ch mixture. By contrast, Ch was found to induce expansion of the DPPE monolayer by 30% at a 1∶1 molar ratio. Strikingly, the DRM-associated lipid, SLPE, did not behave like typical PE but rather like SM, in that a monolayer was compressed when cholesterol had been included in it. Although at 4∶1 SLPE/Ch ideal mixing of the components was observed, higher Ch content caused significant contraction, reaching 10% at 2∶1 and 20% at 1∶1 SLPE/Ch ratio. It is thus comparable in its effect to SM/Ch mixtures in the same conditions.

**Table 1:** Average molecular area vs. composition and percent condensation values for a monolayer measured at a surface pressure of 3 mN/m for 25°C.

Lipid	%Ch	A_i_ [Å^2^]	A_o_ [Å^2^]	% Condensation
**SM**	**0**	65.6	65.6	-
	**20**	58.6	55.0	6.14
	**33**	54.0	46.6	13.80
	**50**	48.2	37.3	22.67
	**100**	30.8	30.8	-
**DPPE**	**0**	55.6	55.6	-
	**20**	50.7	53.9	−6.47
	**33**	47.4	51.0	−7.76
	**50**	43.3	56.2	−30.04
	**100**	30.8	30.8	-
**SLPE**	**0**	66.3	66.3	**-**
	**20**	58.9	59.2	0.46
	**33**	54.0	48.6	10.01
	**50**	47.9	37.9	20.75

As shown above, all studied mixtures displayed deviations from ideality at both low and high surface pressures (see Table 1). The nature (direction) of these deviations differed between raft and non-raft lipids.

**Figure 2:**
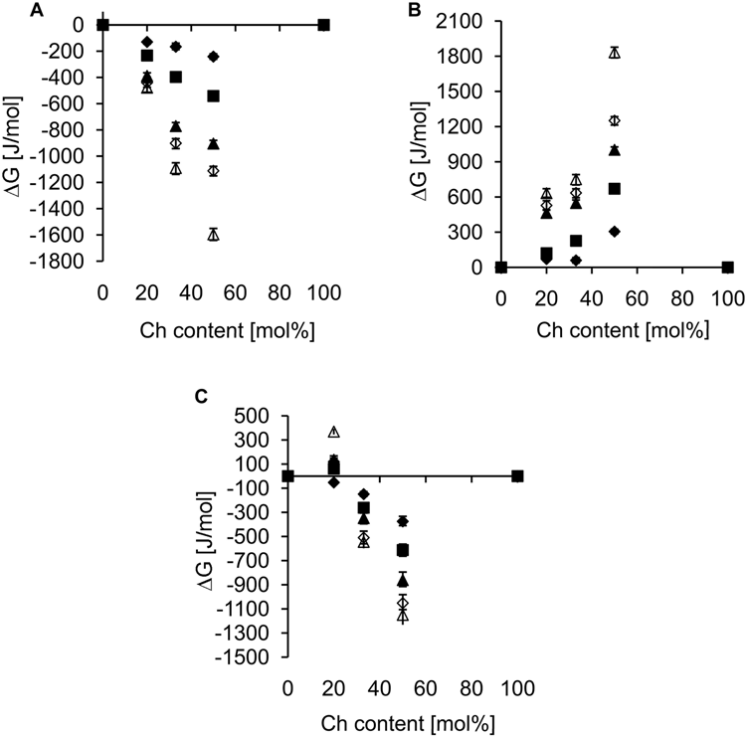
Molar Gibbs excess free energy of mixing as a function of cholesterol content in mixed monolayers. Data for (A) SM, (B) DPPE and (C) SLPE mixtures with cholesterol were obtained at 25°C. Each point represents an average of several experiments. ΔG was calculated at surface pressure of (♦)5, (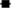)10, (▴)15, (◊)20 and (Δ)30 mN/m.

To quantify the actual interactions between the phospholipids and Ch, the excess free energy of mixing (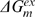) was calculated for the Π-A isotherms for pure and mixed monolayers. The 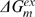 values were calculated at 5, 10, 15, 20 and 30 mN/m and the results are presented in Figure 2. The positive value of 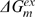 for DPPE/Ch monolayers indicated that the heterologous interactions between Ch and DPPE molecules are weaker than those between the same molecules in monolayers of the separate components. On the other hand, the calculated 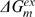 for SM/Ch monolayers is strongly negative, indicating that there are strong interactions between the two components. This would be expected for the raft-forming mixtures. The calculated 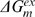 values were also strongly negative for the SLPE/Ch mixtures. Moreover, the interactions between SLPE and Ch appear comparable in magnitude to those recorded for SM/Ch at surface pressure values of 5–20 mN/m. At high surface pressure (30 mN/m), which corresponds to the state in natural membranes, 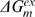 values reach −600 J/mol and more than −1200 J/mol for the 2∶1 and 1∶1 SLPE/Ch monolayers respectively. These values are less negative than those measured for the SM/Ch monolayers at the indicated surface pressure, but are still substantial.

### SLPE-cholesterol interactions are remarkably independent of temperature

Most raft studies were performed at 4°C but the conclusions were taken to apply to structures existing at 37°C. In our studies we tested, whether the specific complex between Ch and SLPE was formed equally at both these temperatures. Therefore, monolayers of Ch, SLPE and 1∶1, 2∶1 and 4∶1 SLPE/Ch mixtures at 4°C and 37°C were formed as described under Materials and Methods. The calculated average molecular areas of SLPE/Ch monolayer at surface pressure of 3 mN/m are presented in Table 2. At both temperatures the SLPE/Ch monolayer was increasingly compressed with increasing proportion of cholesterol, although at low cholesterol content (20 mol%) we observed no specific interactions between SLPE and Ch at 37°C and only a small expansion, reaching 5%, at 4°C. At high Ch contents (33 and 50 mol%) the SLPE/Ch monolayer contracted reasonably. The difference between the level of compression at 4 and 37°C was easily visible but not dramatic (∼3% at both temperatures) indicating that SLPE/Ch interactions remain remarkably independent of temperature.

**Table 2:** Average molecular area and percent condensation values vs. composition of SLPE/Ch mixtures measured at a surface pressure of 3 mN/m at 4 or 37°C.

T [°C]	%Ch	A_i_ [Å^2^]	A_o_ [Å^2^]	% Condensation
**4**	**0**	64.4	64.4	**-**
	**20**	57.8	60.6	−4.93
	**33**	53.4	48.5	9.17
	**50**	47.9	37.3	22.04
	**100**	31.4	31.4	**-**
**37**	**0**	66.0	66.0	-
	**20**	58.6	58.0	0.99
	**33**	53.6	50.3	6.18
	**50**	47.5	38.1	19.73
	**100**	28.9	28.9	-

**Figure 3:**
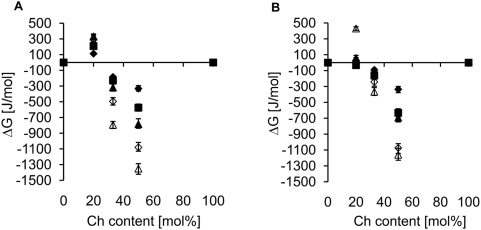
Comparison of ΔG values for SLPE/Ch monolayers at 4 and 37°C. Dependence of molar Gibbs excess free energy (ΔG) of mixing on cholesterol content in mixed cholesterol/SLPE monolayers at (A) 4°C and (B) 37°C. Each point represents an average of several experiments. ΔG was calculated at surface pressure of (♦)5, (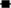)10, (▴)15, (◊)20 and (Δ)30 mN/m.

To learn more about the specific interactions between Ch and SLPE at 4 and 37°C the 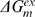 was calculated, as above. Data shown in Figure 3 revealed that at 2∶1 and 1∶1 SLPE/Ch ratios, the interactions between SLPE and Ch were attractive (2∶1 SLPE/Ch) or highly attractive (1∶1 SLPE/Ch), whereas, at low cholesterol concentration (4∶1 SLPE/Ch) a positive 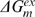 was observed i.e. at 37°C these two monolayer components were almost ideally mixed (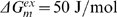, Π = 20 mN/m) while, at 4°C SLPE and Ch interactions could be classified as repulsive (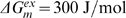, Π = 20 mN/m).

The above data show that a complex of SLPE/Ch was formed at cholesterol concentrations of 33 mol% and above, with only a small temperature dependence (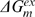 at 4°C and surface pressure of 30 mN/m was only slightly reduced at the lower temperature), while in the remaining cases almost identical values were obtained at all three temperatures (Figure 3).

Our results indicate that the presence of SLPE in DRM fractions was not a chance event or an artefact of isolation, and arised from strong interaction with Ch in membrane.

### Lateral diffusion of NBD-cholesterol in SLPE/Ch complex is similar to those between SM/Ch

**Figure 4:**
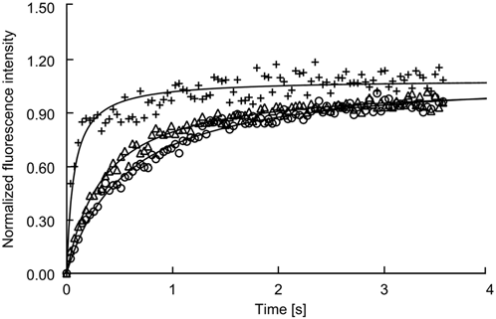
FRAP curves of NBD-cholesterol in SM-/SLPE-/DPPE-based liposomes. Normalized fit curves represent the average values of 10 independent FRAP measurements for each liposome mixture containing 1 mol% NBD-Cholesterol.(○) SM/Ch/DOPC, (Δ) SLPE/Ch/DOPC and DPPE/Ch/DOPC (1∶1∶0.2 molar ratio).

We asked the question whether the lateral mobility of fluorescent probe in SLPE/Ch-based liposomes would be comparable to those formed by SM/Ch vesicles. In Figure 4 the normalized curves of FRAP for SM/Ch/DOPC (1∶1∶0.2), SLPE/Ch/DOPC and DPPE/Ch/DOPC are shown. The parameters obtained from the analysis of experimental data are shown in Table 3. As can be seen the features of SM and SLPE containing liposomes are similar and are characterised by similar curve slope, mobile fraction (R(f) 25 and 20% respectively), diffusion coeficient and τ½ values (see Table 3). The highest diffusion parameters with the relatively low amount of a mobile fraction (∼13%) were obtained for DPPE containing vesicles (see Table 3). This shows that unlike to DPPE/Ch vesicles the parameters obtained for SLPE/Ch liposomes support the concept that there are strong similarities between the properties of the SLPE/Ch and SM/Ch-based bilayers.

**Table 3:** FRAP parameters of NBD-cholesterol in SM-/SLPE-/DPPE-based liposomes.

FRAP Parameters	SM/Ch/DOPC	SLPE/Ch/DOPC	DPPE/Ch/DOPC
D [µm^2^/s]	0.26±0.02	0.53±0.04	1.92±0.09
τ ½ [s]	0.4±0.01	0.27±0.03	0.04±0.01
Recovery [%]	45±0.7	36±1.2	26±1.9

The parameters were obtained as described in Materials and Methods. The data represent mean standard errors of 10 independent experiments. *D* – diffusion coefficient; τ½ – half time.

### TIFF isolation and characterization

**Figure 5:**
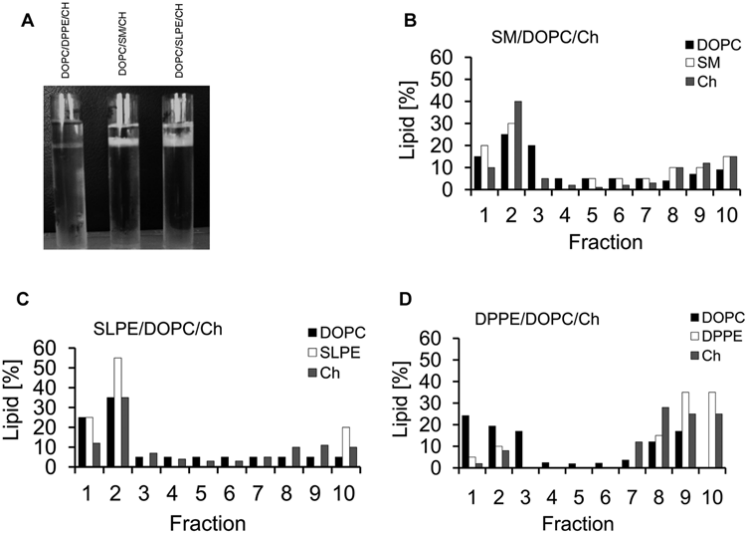
Triton insoluble floating fractions (TIFF) obtained after treatment of liposomes with 1% Triton X-100 containing buffer. (A) Photograph of the gradient samples after centrifugation - TIFFs are observed at the interface between 0–30% sucrose gradient. (B–D) The percentage of particular lipids recovered in fractions collected after fractionation of the gradient samples.

Liposomes consisting of 1/1/1 molar ratios of DOPC/Ch/lipid (where lipid was either DPPE, SLPE or SM) prepared as was described in Materials and methods section were extracted with 1% Triton X-100 and ultrancetrifuged through sucrose gradient. After ultracentrifugation, the Triton Insoluble Floating Fraction (TIFF) was observed in each sample at the interphase of 0%–30% sucrose (Figure 5A). It should be noted however, that in the case of DPPE containing samples the TIFF-coat was quite thin whereas in the SLPE and SM containg samples it was much thicker. The control experiment where, 1% Triton X-100 alone was ultracentrifuged in sucrose gradient did not show any turbidity through all the tube (not shown). Ten fractions were collected from the top of the gradient of each sample (in all cases most of the coat was collected in fraction 2) and the lipids were extracted with chloroform∶methanol and subjected to TLC separation. Lipids were quantified in each spot after staining in iodine vapours as inorganic phosphate and cholesterol as described in Materials and methods. The results are presented as the percentage of particular lipid in the fraction (Figure 5B–D). DPPE was recovered mostly in the bottom fractions (over 80% of DPPE was present in fractions 8–10), whereas majority of SM or SLPE was retrieved in the top fractions (fractions 1–3). It should also be noted that cholesterol was enriched in the top fractions only in the SM and SLPE containig samples what is in agreement with our monolayer results whereas in the DPPE samples cholesterol was recovered in the bottom fractions.

The obtained results suggest that SLPE, similarly to SM, in the presence of high amounts of cholesterol in the membrane can form detergent-resistant phase, therefore its presence in the DRM isolated from natural membranes is most probably not a coincidence.

## Discussion

The lipid raft concept posits that these are well-organized platforms of lipids and proteins floating in the fluid membrane. These structures are thought to have numerous functions [2], [31], [32], [33], [34]. The isolation of DRM, rich in Ch and SM, and the finding that these two lipids formed the l_o_ phase, which is resistant to solubilisation by Triton X-100, became the basis for a model of rafts that could exist in living cells [2], [31], [32], [33]. Although the existence of specific SM/Ch condensed complexes has been a subject of debate being even questioned by several groups e.g.[8] it is almost commonly accepted that these complexes underlay the molecular mechanism of lipid raft formation. One of the biggest drawback of this model was that it confined the rafts to the outer bilayer leaflet, whereas the suggested functions of rafts, such as signal transduction, requires them to be accessible in both leaflets [32]. There were some studies that suggested that transbilayer penetration of long sphingolipid acyl chains might be essential for more highly ordered organization of lipids in the cytoplasmic leaflet [33]. Moreover, it has been shown that lipids can induce fluid-fluid phase separations in the opposing leaflet but only if certain requirements are met [35]. The suggestion that inner-leaflet rafts are enriched in phospholipids with doubly saturated acyl chains was supported by some raft lipidomic studies [20], [23], but efforts to generate the l_o_ phase from inner leaflet lipids have so far been unsuccessful [35]. Moreover, studies with PEs with doubly saturated acyl chains concluded that these lipids could not form any specific complexes with Ch [33]. On the other hand, Ch has been shown to have strong affinity for POPE that is comparable to SM [27] and at high levels of Ch in POPE a l_o_ phase can be formed [29]. Here we showed that (i) monolayers of SLPE, which is more abundant in rafts, was progressively compressed on incorporation of increasing proportions of Ch; (ii) SLPE was able to form a specific complex with Ch; and (iii) the strength of the interactions SLPE with Ch was comparable to that reported for Ch with SM.

In our studies we used monolayers as a model of membrane lipid interactions. This is a widely used method that offers the advantage of reducing the complexity of natural membranes [36]. In the first place, we compared the interactions of SM, DPPE and SLPE with Ch at 25°C. SM/Ch interactions were taken as an example of possible interactions that could occur in rafts, and DPPE, with its two saturated acyl chains, was chosen as a representative of PEs. For comparison of the interactions between these lipids, the monolayer contractions and 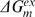 values were determined. SM and DPPE in the presence of Ch behave in opposite ways, the first causing compression with increasing Ch content with a significant negative value of 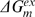, while DPPE films expanded in the presence of cholesterol. The positive values of 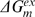 in this case confirm that no specific complex between these two components was formed (Figure 2). Our data are in good agreement with the studies on PE and Ch [27], [28], [29], but are in conflict with another report, describing compression of DPPE films in the presence of Ch [25]. It should be noted, though, that this effect was independent of the Ch content of the monolayer, and that the reported contraction was only ∼8%. In our studies with SLPE/Ch mixtures, a much higher compressing effect was observed, reaching 21% for 1∶1 SLPE/Ch and ∼10% for 2∶1 SLPE/Ch monolayers. Such a large effect was similar to that observed in SM/Ch mixed monolayers (Table 2). To quantify the strength of the SLPE/Ch interaction, 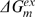 was calculated. The negative values confirmed that the observed interactions are highly specific and are only slightly weaker than those found for SM/Ch, reaching ∼−1250 J/mol, as compared to ∼−1500 J/mol for SM/Ch (Figure 2). It therefore appears beyond doubt that there is a specific interaction between SLPE and Ch.

Rafts are suggested to exist in the plasma membrane of normal living cells, in mammals at 37°C. Yet the methods of isolating DRM, which are very often regarded as rafts, specify a temperature of 4°C, and this may be the cause of many misleading observations and artefacts. To avoid these, we measured isotherms of SLPE/Ch mixed monolayers at 4°C and at 37°C. The resulting area changes and 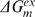 values (Figure 3) show that both were almost independent of temperature at 1∶1 and 2∶1 SLPE/Ch ratios. Although at 1∶1 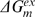 was lower at 4°C (−1450 J/mol) than at 37°C (−1250 J/mol), this small difference does not allow us to conclude that the specific complex forms only at 4°C, but rather that there is a strong possibility of its existence at both temperatures. The noticeable difference occurs in the 4∶1 SLPE/Ch monolayers, where repulsive interactions were observed at 4°C at all surface pressures, while at 37°C this was seen only at a pressure of 30 mN/m. For the remaining calculated surface pressures, ideal mixing of SLPE with Ch was observed at 37°C. It is important to note that with increasing surface pressure 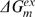 became increasingly negative, reaching more than −1200 J/mol at 30 mN/m. This surface pressure corresponds to the state of the lipids in native membranes, and the negative 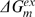 at 37°C suggests that such complexes can indeed form in physiological conditions.

We show here that DPPE does not form complex with Ch. This is in agreement with other studies, that present that although in DPPE/Ch mixtures phase separation is observed this effect is rather caused by strong Ch/Ch and DPPE∶DPPE interactions [25]. Saturated PE species do not form complexes with Ch which is in contrast to saturated PC species, which were found compressed in the presence of Ch and the 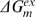 of the interactions was strongly negative [17]. Monounsaturated POPE and POPC also differ in this respect. In SM/Ch/POPE mixtures no phase separation was observed whereas SM/Ch/POPC is a commonly used mixture for the studies of l_o_/l_d_ phase separation [11], [17], as the affinity of Ch to SM is several fold larger compared to POPC [33]. It is then interesting that the interactions of both saturated (DSPC) and unstaurated (SOPC and DOPC) with Ch were characterized by strongly negative 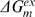 [17], which means that both lipids formed tight complexes with Ch. Therefore, the enrichment of SOPC in DRM [20] is fully understood. Although PCs are mainly recognized as the outer leaflet lipid, it should be remembered that some part of this lipid species are present also in the inner leaflet (∼15% of the total inner leaflet lipids depending on the membrane). Although in this case, the probability of the existence of PC/Ch complexes as the major component of the inner monolayer of membrane rafts is rather small. In contrast to PCs, the interactions of PEs with Ch change from highly repulsory for saturated DSPE [25] or DPPE to attractive for monounsaturated POPE [27] and highly attractive for SLPE, as presented here. It would seem that the affinity of Ch for PE species increases with level of unsaturation of PE acyl chains, however, PDPE (1-palmitoyl-2-docosahexaenoyl-sn-glycerophosphatidylethanolamine) which has a polyunsaturated acyl chain was shown to have rather poor affinity for Ch [27]. The poor affinity of Ch for polyunsaturated acyl chains was proposed previously to trigger raft formation within membranes [11], [26].

The l_o_ phase is described as being bridge between the gel and fluid lamellar phases, depending on the temperature and the cholesterol content of the system. One of the characteristics is the lateral mobility of the molecules in the l_o_ phase [37]. Our FRAP experiments (Figure 4) suggest that the lateral mobility parameters of NBD-Ch in SLPE/Ch liposomes are closer to those of SM/Ch than to DPPE/Ch liposomes. D and *τ½* (Table 3) for for SM/Ch and SLPE/Ch vesicles differ less than twice. As one would expect the lowest mobility of the fluorescence probe was obtained for the well defined order-like domains composed of SM/Ch vesicles. In such liposomes the environment is mostly predominated by highly ordered and stiffness interactions formed between SM/Ch in which mobility of fluorescent tracer is partialy reduced. On the other hand considerably high mobility of fluorescence probe in DPPE/Ch membrane might reflect the weak affinity of DPPE to Ch that probably forced the phase separation between DPPE/DPPE molecules with simultaneously repulsion of Ch into Ch/Ch clusters. Even though in such conditions most of the probe molecules might be trapped in the immobile-like cholesterol clusters and the fast recovery fraction dramatically decreases (∼R(f) 13%), the mobility of fluorescence probe is still reasonably higher than in the raft-like vesicles. Unlike the DPPE/Ch the diffusion coefficient obtained for SLPE/Ch mixtures and other calculated FRAP parameters were much closer correspond to those obtained for SM/Ch mixtures, confirming suggestion that the interactions between SLPE/Ch occurred. Importantly our results are in agreement with the other groups working on diffusional behaviour of different fluorescent probes in raft-like domains as the diffusion coefficient values for l_d_-like and l_o_-like mixture bilayers differ by not more than one order of magnitude [38], [39]. In should be mentioned that, comparison of the data obtained from the measurements of the area fractions of the l_o_ phase in planar supported bilayers with the areas of l_o_ phases in multilamellar liposomes composed of PC/SM/Ch by using different techniques (direct visualisation, FRET analysis) yielded the same percentage area fraction for both (∼60%), suggesting that liposomes might also be successfully employed to measure changes of the diffusional mobility of the fluorescence probes upon phase separation in different conditions [39]. It should be taken into account though, that the attachment of NBD into the acyl chain of Ch seems to generally decrease the affinity of this lipid for the more ordered domains as it was shown by AFM correlated fluorescence microscopy studies, where sorting out of NBD-Ch (2 mol%) from membranes existing in a cholesterol-rich phase was observed in DOPC/SM/Ch supported planar bilayers [40]. On the other hand it has been recently demonstrated that the ability of NBD-Ch to partition into the gel-like membranes highly depends on its concentration (≥1 mol%) and structural orientation in the membrane [41]. Nevertheless, according to our findings the goal of our studies was to estimate whether the latteral mobility of fluorescently labeled probe would be similar in SLPE/Ch domain compared to SM/Ch raft-like domains and opposite to those composed of DPPE/Ch.

Further evidence on the nature of SLPE/Ch interactions comes from the Triton solubilization experiments. It has been shown before that the l_o_ phase consisting of SM/Ch can be isolated from the membranes as the TIFF [42], Here we performed the solubilization of the membranes consisting of DOPC/Ch/lipid (where lipid was either DPPE, SLPE or SM). Our observations, revealed that SLPE in the presence of high amounts of Ch in the membrane was even more resistant to solubilization than the SM/Ch complex (Figure 5). However, it should be remembered that we used egg SM, which is a mixture of various SM species with various lengths and degree of saturation of the acyl chain. Such result may suggest that SLPE/Ch forms most probably a l_o_ phase similar to the one observed for SM/Ch mixtures, what in turn implicates that various other unsaturated PE species may in fact form complex with Ch that restrain solubilisation with cold nonionic detergents during DRM isolation from natural membranes.

Several studies of the lipid composition of rafts have shown that Ch and SM are almost equimolar in DRM [21], [22], [23], [24]. It may also be noted that DRM preparations observed by using freeze fracture technique in the electron microscope show that rafts are bilayer structures, although it is not known whether the lipid asymmetry is preserved during the isolation of DRM [43]. However, keeping in mind that the bulk of SM is present in the outer bilayer leaflet and that Ch is more or less equally distributed between the leaflets, there is a probability that 50% of Ch in DRM derives from the inner leaflet. Therefore we cannot exclude that some inner leaflet phospholipids, such as PE's containing double unsaturated acyl chains in sn-2 position, form complexes with Ch, comparable to those between SM and Ch. Our attention should also be paid to the abundantly present in DRM preparations PE plasmalogens [18], [22]. So far, no specific explanation for their enrichment in the DRM was proposed. It has been suggested that the key to inner leaflet l_o_ phase organization is actually the transbilayer penetration of long sphingolipid acyl chains [33], [35], however, the inner leaflet membrane lipid domains could still consist of lipid/Ch complexes. Our results support the hypotesis that small rafts existing in the plane of the biomembranes are driven by the tendecies of ceratin lipids to interact. Here we demonstrated that SLPE, a DRM-associated lipid, may form such complexes with Ch. Moreover, these interactions were almost as strong as those found for SM/Ch and were stable not only at the temperature of raft isolation but also at 37°C, at which rafts should exist in living cells.

## Materials and Methods

### Materials

Egg sphingomyelin (SM) was from Lipid Products; 1,2-Dipalmitoyl-sn-Glycero-3-Phosphoethanolamine (DPPE) and 1-Stearoyl-2-Linoleoyl-sn-Glycero-3-Phosphatidylethanolamine (SLPE) which is 1-Stearoyl-2-*cis,cis*-Δ^9^,Δ^12^Octadeca-dienoyl-sn-Glycero-3-Phosphatidylethanolamine were from Avanti Polar Lipids; Cholesterol (Ch) was from Northern Lipids. Lipid concentrations were quantified by phosphate analysis [44]. Cholesterol concentration was quantified either by the method of Courchaine et al. [45] or with Amplex Red Kit (Invitrogen).

### Monolayers

Monolayers were prepared as described previously [46] with a few modifications. Briefly, chloroform solutions of pure lipids (DPPE, SLPE, SM, Ch) or mixtures 4∶1, 2∶1 and 1∶1 of DPPE/Ch, SLPE/Ch or SM/Ch were prepared prior to injecting into the subphase (deionized water, pH 7.0). Each monolayer was prepared by injecting up to 10 µl of the lipid solution onto the subphase to give a mean area per molecule of 95±3 Å^2^. The isotherms were recorded using a 70 cm^2^ teflon Langmuir trough fitted with a motorized compression barrier (Nima Technology) equipped with a pressure sensor and Wilhelmy plate. The trough was surrounded by a water jacket supplied by a temperature-controlled, circulating water bath. The isotherms were recorded at 25°C and for the SLPE/Ch mixtures additional isotherms were recorded at 4°C and 37°C. The trough was placed in a chamber facilitating flushing with nitrogen to avoid oxidation of the lipids. The barrier speed was set at 30 cm^2^/min. For each lipid mixture at least 3 independent monolayers were prepared and for each monolayer 10–12 isotherms were recorded. For the analysis the 5–7 most consistent isotherms from each monolayer were chosen.

### Data analysis

Area/lipid molecule was read at surface pressure Π = 3 mN/m, as an alternative to assessing area/molecule at low surface pressure and extrapolating to zero pressure [47]. The results were used to calculate the theoretical mean area per molecule for non-interacting molecules as follows:
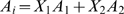
(1)where: *A_i_* – the mean molecular area, *X_1_*, *X_2_* – mole fraction of component 1 or 2, *A_1_* and *A_2_* – the mean molecular areas of pure components 1 or 2 at surface pressure Π = 3 mN/m [26].

The percent molecular area change (compression) was calculated as follows:

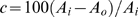
(2)where: *c* – % of compression, *A_o_* – the observed molecular area at Π = 3 mN/m, *A_i_* – the theoretical mean molecular area of two non-interacting molecules.

To demonstrate mixing of molecules in the monolayers the excess free energy of mixing was calculated [48],
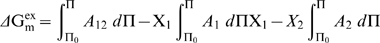
(3)where: 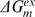 free excess energy of mixing, X – mole fraction of components, Π_0_ = 0 mN/m, Π = 5, 10, 15, 20 or 30 mN/m, A – area of a single molecule (Å^2^), The integrals from Π->A were calculated in MS Excel by using a modified Reimann sum.

### MLV preparation for FRAP experiments

The multilamellar vesicles consisted of SM//Ch/DOPC, SLPE/Ch/DOPC or DPPE/Ch/DOPC (1∶1∶0.2 molar ratios) containing 1 mol% NBD-Cholesterol were prepared by mixing required amount of the chloroform lipid solutions, dried the solvent by evaporation and keeping the films under vacuum for 3–4 h in the dark. The lipid films were hydrated with PBS (137 mM NaCl, 3 mM KCl, 6 mM Na_2_HPO_4_, 2 mM KH_2_PO_4_, pH 7.4) and allowed to rehydrate at 37°C for 30 min with intermittent swirling. The vesicles were kept at 4°C in the dark for two days. The final concentration of lipids in liposome suspensions was 2 mg/ml. An aliquot (∼10 µl) of each liposome solutions were sandwiched between glass slide and a coverslip, the edges of the coverslip were sealed with silicone and used for FRAP analysis.

### FRAP analysis

All FRAP experiments were made with a Zeiss LSM510 confocal scanning microscope (Jena, Germany). Images were acquired with a 63×, 1.2NA water-immersion objective using 488 nm line of argon laser as the excitation source. Fluorescence emmision of NBD-cholesterol were detected using the control of an acusto optical tuneable filter (AOTF). Images were acquired with a pinhole set to 1 Airy unit at a 512×64 pixel resolution (0.04 µm/pixel). Photobleaching of NBD-cholesterol was performed by using 120 scans (max speed 13, 0.64 µsec/pixel) with the 488 nm laser line at full power in a circular region of interest (ROI) defined as a circle of 1.44 µm in diameter. Pre- and postbleach scans were monitored at low laser intensity. Fluorescence recoveries during the time series were quantified using Zeiss LSM510 software (ZEN 2007). All experiments were performed at 23°C. Statistical analysis using F-test was carried out with Microsoft Excel software. The fluorescence recovery kinetics was analysed to determine the characteristic diffusion time (τ_d_) on the basis of a model describing fluorescence recovery into a uniformly bleached circular disc [49]:
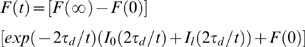
(4)Where *F(t)* is normalized fluorescence intensity at time t in the circular region of interest, *F(∞)* is the recovered fluorescence intensity at time t(∞), *F(0)* is the bleached fluorescence intensity at time t(0), *I_0_* and *I_l_* are modified Bessel functions. The bleached time point was calculated as a mid-point of the bleach duration. This resulted in the first post-bleached time point starting from time t>0. Nonlinear curves fitting of the fluorescence recovery data to equation (4) were carried out by using Zeiss ZEN software. The diffusion coefficient *(D)* was determinated from the equation:
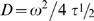
(5)Where *ω* is the radius of circular ROI, *τ*½ diffusion half time. The mobile fraction *R(f)* that represent the fraction of probe available for diffusion were defined as follows:
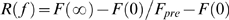
(6)Where *F_pre_* is the fluorescence intensity of the area before bleaching.

### Triton insoluble floating fraction (TIFF) isolation and TLC evaluation of lipids

Unilamellar vesiscles (∼100 nm) were prepared by mixing chloroform solutions of appropriate compounds, evaporation of the solvent under stream of nitrogen and then under vacuum over night. The lipid films were hydrated with Tris-HCl buffer (10 mM Tris-HCl, 150 mM NaCl, 5 mM EDTA, pH 7,5) and the resulting vesicles were calibrated through 100 nm pore membranes (Whatman) and their size was checked in a Malvern ZetaSizer. The final concentrations of lipids in the liposome solution was 2 mg/ml, and the liposomes consisted from DPPE/DOPC/Ch, SLPE/DOPC/Ch or SM/DOPC/Ch (1∶1∶1 molar ratios). After extrusion the vesicles were pooled by ultracentrifugation (2 h, ∼100 000×g, Beckman 60Ti swinging bucket rotor). After ultracentrifugation the vesicles were suspended in 300 µl cold 1% Triton X-100 containing buffer (1% Triton X-100, 10 mM Tris-HCl, 150 mM NaCl, 5 mM EDTA, pH 7.5). The samples were left on ice for 20 minutes and were vigourosly vortexed occasionally. Subsequently samples were mixed with equal volume of 80% sucrose, overlaid with 3200 µl of 30% sucrose and 500 µl of Tris-HCl buffer. Samples were ultracentrifuged as above, and 400 µl fractions were collected from the top of the gradient. The fractions were extracted with chloroform∶methanol (3∶1) solutions, and the organic phase containing lipids was separated using TLC technique (chloroform∶methanol∶acetic acid∶water 25∶15∶8∶2 as a moving phase was used). The lipids were visualized using iodine vapours. The spots containing separated lipids were scrapped, collected into test tubes and extracted three times with chloroform∶methanol (3∶1). Lipid and cholesterol concentrations were quantified as described above.
